# Visualizing the modulation of neurokinin 1 receptor-positive neurons in the superficial dorsal horn by spinal cord stimulation *in vivo*

**DOI:** 10.1097/j.pain.0000000000003361

**Published:** 2024-08-13

**Authors:** Qian Xu, Qin Zheng, Xiang Cui, Andrew Cleland, Juan Hincapie, Srinivasa N. Raja, Xinzhong Dong, Yun Guan

**Affiliations:** a The Solomon H. Snyder Department of Neuroscience, Johns Hopkins University, School of Medicine, Baltimore, Maryland, 21205, USA.; b Howard Hughes Medical Institute, Johns Hopkins University, School of Medicine, Baltimore, Maryland, 21205, USA.; c Department of Anesthesiology and Critical Care Medicine, Johns Hopkins University, School of Medicine, Baltimore, Maryland, 21205, USA.; d Medtronic, Inc, Minneapolis, MN, USA.; e Department of Neurological Surgery, Johns Hopkins University, School of Medicine, Baltimore, Maryland 21205, USA.

**Keywords:** spinal cord stimulation, calcium imaging, neurokinin 1 receptor, dorsal horn, neuropathic pain, mice

## Abstract

Spinal cord stimulation (SCS) is an effective modality for pain treatment, yet its underlying mechanisms remain elusive. Neurokinin 1 receptor-positive (NK1R^+^) neurons in spinal lamina I play a pivotal role in pain transmission. To enhance our mechanistic understanding of SCS-induced analgesia, we investigated how different SCS paradigms modulate the activation of NK1R^+^ neurons, by developing NK1R-Cre;GCaMP6s transgenic mice and employing *in vivo* calcium imaging of superficial NK1R^+^ neurons under anesthesia (1.5% isoflurane). NK1R^+^ neurons in the lumbar spinal cord (L4–5) showed a greater activation by electrical test stimulation (TS, 3.0 mA, 1 Hz) at the hindpaw at 2 weeks after tibia-sparing nerve injury (SNI-t) than in naïve mice. SCS was then delivered through a bi-polar plate electrode placed epidurally at L1–2 level. The short-term 50 Hz high-intensity SCS [80% motor threshold (MoT), 10 min] induced robust and prolonged inhibition of NK1R^+^ neuronal responses to TS in both naïve and SNI-t mice. The 30-minute 50 Hz and 900 Hz SCS applied at moderate intensity (50% MoT) also significantly inhibited neuronal responses in SNI-t mice. However, at low-intensity (20% MoT), the 30-minute 900 Hz SCS only induced persistent neuronal inhibition in naïve mice, but not in SNI-t mice. In conclusion, both 10-minute high-intensity SCS and 30-minute SCS at moderate intensity inhibit the activation of superficial NK1R^+^ neurons, potentially attenuating spinal nociceptive transmission. Furthermore, *in vivo* calcium imaging of NK1R^+^ neurons provides a new approach for exploring the spinal neuronal mechanisms of pain inhibition by neuromodulation pain therapies.

## 1. Introduction

Neuropathic pain poses a significant challenge to treat with existing pharmacotherapies[15]. Spinal cord stimulation (SCS), an alternative therapeutic strategy, has demonstrated clinical effectiveness and safety for mitigating various pain conditions. This is especially evident with the advent of new paradigms and technologies, such as Differential Target Multiplexed (DTM) SCS[9; 21], burst SCS and high-frequency SCS[12; 21; 28; 39; 60]. Despite these advancements, the mechanisms that contribute to the pain-inhibitory effects of different SCS therapy paradigms remain incompletely understood. This knowledge gap hinders further improvement in the efficacy and accessibility of SCS[21; 39].

Spinal neuronal mechanisms are essential to pain inhibition resulting from SCS[2; 19; 39; 50]. Diverse types of primary sensory neurons are responsible for detecting and transmitting different modalities of sensory information from the periphery to the spinal cord. Among these, the majority of unmyelinated afferents (C-fibers) are thermoreceptors or nociceptors, which detect and transmit pain signals. Their central processes mostly terminate in the superficial dorsal horn, a region critical to spinal nociceptive transmission and modulation[6; 7]. This region houses important spinal projection neurons, interneurons, and descending nerve terminals from the brain, collectively forming neuronal circuitries that fine-tune the ascending pain transmission. In lamina I, only a small proportion of neurons are spinal-brain projection neurons, and they are dispersed throughout the area[57]. Strikingly, previous studies have suggested that a significant portion of these projection neurons (~ 80%), which play a pivotal role in the transmission of noxious stimuli, are positive for neurokinin 1 receptor (NK1R^+^)[48; 54; 57]. However, the role of superficial NK1R^+^ neurons in SCS-induced pain inhibition remains unclear. This is partly due to significant challenges associated with using traditional electrophysiologic methods to efficiently identify these neurons and maintain a stable recording while surveying their activity *in vivo*.

Beyond the generation of action potentials, neurons exhibit robust calcium-based excitability and intracellular calcium transients in response to stimulation[22]. The use of a Genetically Encoded Calcium Indicator (GECI) known as GCaMP allows for monitoring and quantification of increases in cytosolic calcium concentration, as indicated by the rise in fluorescence intensity in calcium imaging. This method also enables the tracking of the activities of a population of cells over extended periods at single-cell resolution[20; 47]. Accordingly, we have generated NK1R-Cre;GCaMP6s transgenic mice, which allow for the visualization of the calcium responses of superficial NK1R^+^ neurons *in vivo* without ambiguity. By using the two-photon imaging, we examined the modulatory effects of low-rate (50 Hz) and high-rate (900 Hz) SCS at different durations and intensities on superficial NK1R^+^ neurons during spinal pain transmission *in vivo*, aiming to enhance our understanding of the mechanisms underlying pain inhibition from different SCS paradigms under neuropathic pain conditions.

## 2. Methods

### 2.1. Animals

**Figure 1.**
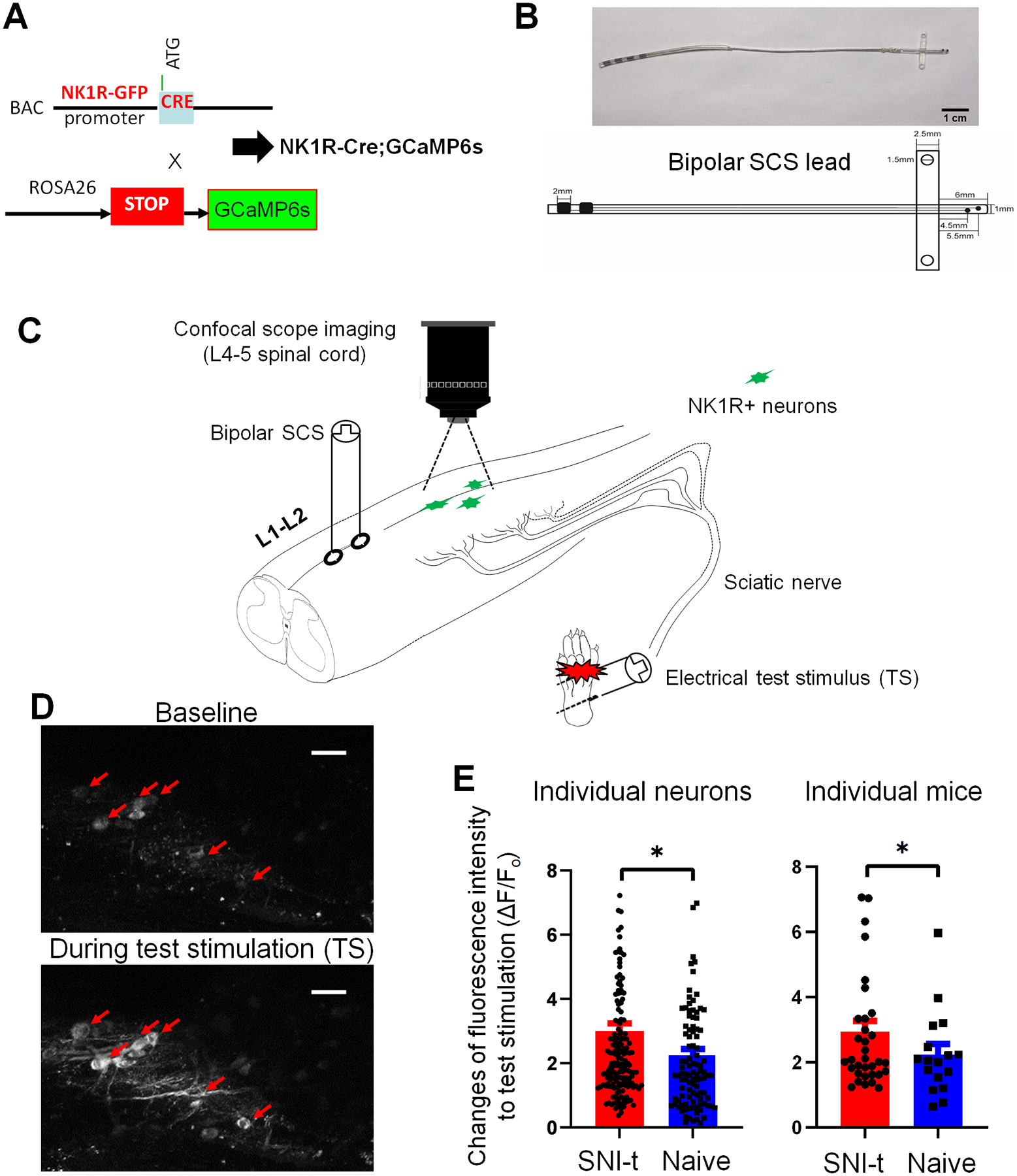
Experimental setup and protocol. **(A)** Strategy for generating NK1R-Cre;GCaMP6s mice. **(B)** The miniature bipolar SCS lead for mice (Medtronic, Minneapolis, MN). **(C)** Schematic diagram illustrating two-photon microscopy setup for imaging spinal dorsal horn neurons in anesthetized NK1R-Cre;GCaMP6s mice. The SCS lead was placed epidurally over the cord at the L1–L2 level. *In vivo* calcium imaging of L4–5 spinal segments was conducted with a Scientifica Galvo Multiphoton System (Scientifica inc.). After stable baseline recording, the plantar side of hindpaw was stimulated with intracutaneous electrical test stimulation (TS, 3.0 mA, 2.0 ms, 1 Hz, 10 sec, biphasic, constant current) by a pair of needs during live imaging. **(D)** Examples of calcium responses in NK1R^+^ neurons (red arrow) to TS, scale bar: 50 μm. Please also see the supplemental video. **(E)** Calcium responses in NK1R^+^ neurons to TS in SNI-t (n=175 neurons from 34 mice) and naïve mice (n=103 neurons from 17 mice). Evoked calcium signal amplitudes to TS were expressed as ΔF/F_o_, data were plotted as individual neurons (left) and individual animals (right). Data are presented as mean ± SEM. *P<0.05, unpaired t-test.

NK1R-Cre;GCaMP6s transgenic mice were generated by cross-breeding NK1R-GFP-Cre with *Rosa26-loxP-STOP-loxP-GCaMP6s* mice (Fig. 1A). The NK1R-GFP-Cre mouse line was generated by using a homologous recombination knock-in approach. The GFP-Cre transgene was integrated into the start codon of the NK1R gene using two homologous arms targeting the sequences of TTTGCTGCCTTGCCGCAAAATG and CTGAAAATTAAGAAAGTGCCC to replace the entire NK1R coding sequence. ROSA26LSL-GCaMP6s mouse lines were acquired from Dr. Bergles’ laboratory at Johns Hopkins. These transgenic mice were backcrossed to C57BL/6J mice for at least 10 generations. All NK1R-Cre;GCaMP6s transgenic mice used in current study were heterozygous and NK1R is functionally expressed. Mice were housed in groups of three to five on a standard 12-hour light/12-hour dark cycle with free access to food and water. All use of animals was approved by the Animal Care and Use Committee of Johns Hopkins University and complied with the National Institutes of Health *Guide for the Care and Use of Experimental Animals* to ensure minimal animal use and discomfort. The transgenic animals created in our lab are available to the broader research community upon receipt of justified request.

### 2.2. Tibia-sparing nerve injury (SNI-t) model of neuropathic pain

Young adult mice (4–6 weeks, both sexes) were anesthetized with isoflurane delivered through a nose cone (2.0%) and breathed spontaneously throughout the surgery. The skin on the leg was shaved and cleaned with a 10% povidone–iodine solution. Under aseptic conditions, the left side of the sciatic nerve was exposed at the mid-thigh level by blunt dissection through the biceps femoris muscle. Using a modified method described in previous studies[5; 13; 27], nerve injury was produced by an 8–0 nonabsorbable sutures silk with tight ligation of the two branches of the sciatic nerve, the common peroneal nerve, and the sural nerve, followed by the transection and removal of about 1–2 mm nerve portion. The tibial nerve, which innervates the medial portion of the hindpaw, was kept intact so that stimulation could be applied to the mid-plantar area of the hindpaw to test pain responses. Hemostasis was achieved. The muscle layer was closed with 6–0 Polysyn^™^ (synthetic absorbable) sutures, and the skin was closed with metal clips. No surgery was performed on the contralateral side (right). Animals were returned to their cages after the surgery, kept warm under a heat lamp, and monitored during recovery. Skin staples were removed around 7 days post-surgery while the animal was under brief gas anesthesia delivered through a nose cone (isoflurane, 2%).

### 2.3. Spinal cord stimulation (SCS)

A small bi-polar plate electrode for SCS in mice was developed by Medtronic Inc. (Fig. 1B). Calcium imaging was conducted in naïve NK1R-Cre;GCaMP6s mice, and those at two weeks after SNI-t, which is the typical time required for the full development of neuropathic pain-like behavior in this model[16; 27; 38; 63]. On the day of calcium imaging, the miniature bipolar SCS electrode was acutely placed epidurally at spinal segments (L1-L2), which are rostral to the imaging segment (L4–5, Fig. 1C), following a dorsal laminectomy. Table 1 shows the parameters for different paradigms of SCS or sham SCS (sham) in each group. Upon the completion of the experiments, the mice were humanely euthanized. The SCS electrode was subsequently extracted, thoroughly cleaned, inspected for electrical connectivity and stored for future use.

Two protocols were used for SCS: 1) Two 10-minute sessions of conventional SCS with a low-rate (50 Hz) but high-intensity (80% MoT) were applied, and a test stimulation was conducted between the sessions (Protocol I). The goal was to determine whether the second session could induce a greater or cumulative inhibitory effect than the first, immediately after completing each short SCS. 2) Given that high-frequency SCS has been shown to achieve pain inhibition at lower intensities (e.g., < 50% MoT) than conventional SCS (e.g., 80% MoT) in animal studies[14; 41; 42], but may require a longer treatment duration[2; 21; 36], we applied a single 30-minute session of 900 Hz SCS (Protocol II) at either moderate (50% MoT) or low (20% MoT) intensities in SNI-t mice.

### 2.4. Electrical test stimulation

During calcium imaging, electrical test stimulation (TS, 3.0 mA, 2.0 ms, 1 Hz, 10 seconds, biphasic, constant current) at an intensity that is high enough to activate both A- and C-fibers was delivered through a pair of needles inserted into the mid-plantar area of the left hindpaw, which is ipsilateral to the side of nerve injury.

### 2.5. Motor threshold (MoT) determination

The intensity of SCS was determined by observing MoT to 4 Hz stimulation (0.15 ms pulse width, biphasic, constant current,) in the lower-limb and low-back area in the anesthetized condition with isoflurane (1.5%), as described in our previous studies[14; 41; 59]. The individual MoT values were measured for each animal, allowing us to tailor the intensity of SCS according to the specific MoT of that particular animal. Supplemental Fig. 1 illustrates an example of MoT measured in a subgroup of mice.

### 2.6. Two-photon imaging

Naïve NK1R-Cre;GCaMP6s mice and those at 2 weeks after SNI-t surgery were deeply anesthetized with an intraperitoneal injection of pentobarbital (50 mg/kg, i.p.) during surgical preparation. Anesthesia was then transited and maintained with isoflurane (1.5%) delivered through the nose cone throughout the imaging and testing phase. Two-photon imaging of dorsal spinal cord was conducted as described in our previous study[57]. The lumbar spinal cord (L4–5) was exposed by dorsal laminectomy from the T10-L1 spinal vertebrate level (Fig. 1C). The dura mater remained intact and 3% agarose was used to create a small well in which to place an Olympus 20× water-immersion objective, bathed in ACSF. Then we carefully rotated the animal around the longitudinal axis by approximately 30 degrees for imaging with the Scientifica Galvo Multiphoton System and Coherent Chameleon Ultra II laser.

The laser was tuned at 900 nm for two-photon excitation for GCaMP6 and the laser power was set to the lowest level (~20 mW) to avoid phototoxicity. We conducted XYZT imaging. The imaging process involved scanning at a rate of 10 frames for each Z cycle, with each cycle having a duration of 10 seconds. The inherent galvo-scan rate was set at 1 frame per second, and each Z-cycle was composed of 10 planes. The acquisition pattern adhered to a stair-step method, where each plane was sampled once prior to transitioning to the subsequent plane. This resulted in an effective sampling rate of 0.1 Hz. To determine the distance between planes, we initially identified the neuron’s surface and then proceeded with imaging in a downward direction to encompass the entire neuron, segmenting this range into 10 frames. The image resolution was 512 × 512 pixels for scanning a larger area. For the duration of the imaging experiment, the body temperature of the mice was maintained at 36.0–37.0°C with a heating pad, which kept the corresponding skin temperature at 35.0°C, as monitored by a probe placed under the abdomen.

### 2.7. Quantification of calcium imaging

Quantification of calcium imaging data was conducted as described in our previous studies[20; 57]. To assess the effects of SCS on evoked calcium responses of dorsal horn neurons, we recorded and exported raw calcium imaging data as Tagged Image File Format (TIFF) files. Responses of neurons to TS were examined before and after SCS or sham stimulation. We exported the raw images (TIFF) and used ImageJ (National Institutes of Health) to analyze calcium imaging data. To address movement-induced image shift and ensure consistent analysis of the same cells throughout the recording, we used StackReg plugin (http://bigwww.epfl.ch/thevenaz/stackreg/) for drift correction. The StackReg plugin registers a stack of image slices by aligning each slice to the next and also requires the installation of the TurboReg plugin (http://bigwww.epfl.ch/thevenaz/turboreg/). To correct for shifts in XY and Z planes over time (T), we first grouped the ten Z-frames into one frame, effectively transforming our data into a new XYZ image stack where the Z dimension represents time (previously T). We then applied StackReg to align these images. By aligning each slice to the next, StackReg can correct for drift in both the XY and Z dimensions. Once aligned, consistent Regions of Interest (ROIs) can be defined manually, saved, and reloaded for each time point. The Time Series Analyzer plugin can then track and quantify changes within these ROIs across the entire stack. We have used this same method in previous studies[57; 64]. Calcium responses were assessed as the increase in green fluorescence intensity of the GCaMP upon binding to intracellular calcium. The response was quantified as the maximum change in fluorescence during the 10-second test stimulation period. Fluorescence intensity at the baseline level without any stimulation was taken as F_0_. Evoked calcium signal amplitudes to test stimulation (F) were expressed as ΔF/F_o_, a ratio of the fluorescence difference (ΔF=F-F_o_) to basal fluorescence. Activation of a cell was defined as an increase in fluorescence intensity (ΔF) ≥ 30% of baseline (F_0_), as shown in previous studies[20; 57].

### 2.8. Statistical analysis

**Figure 2.**
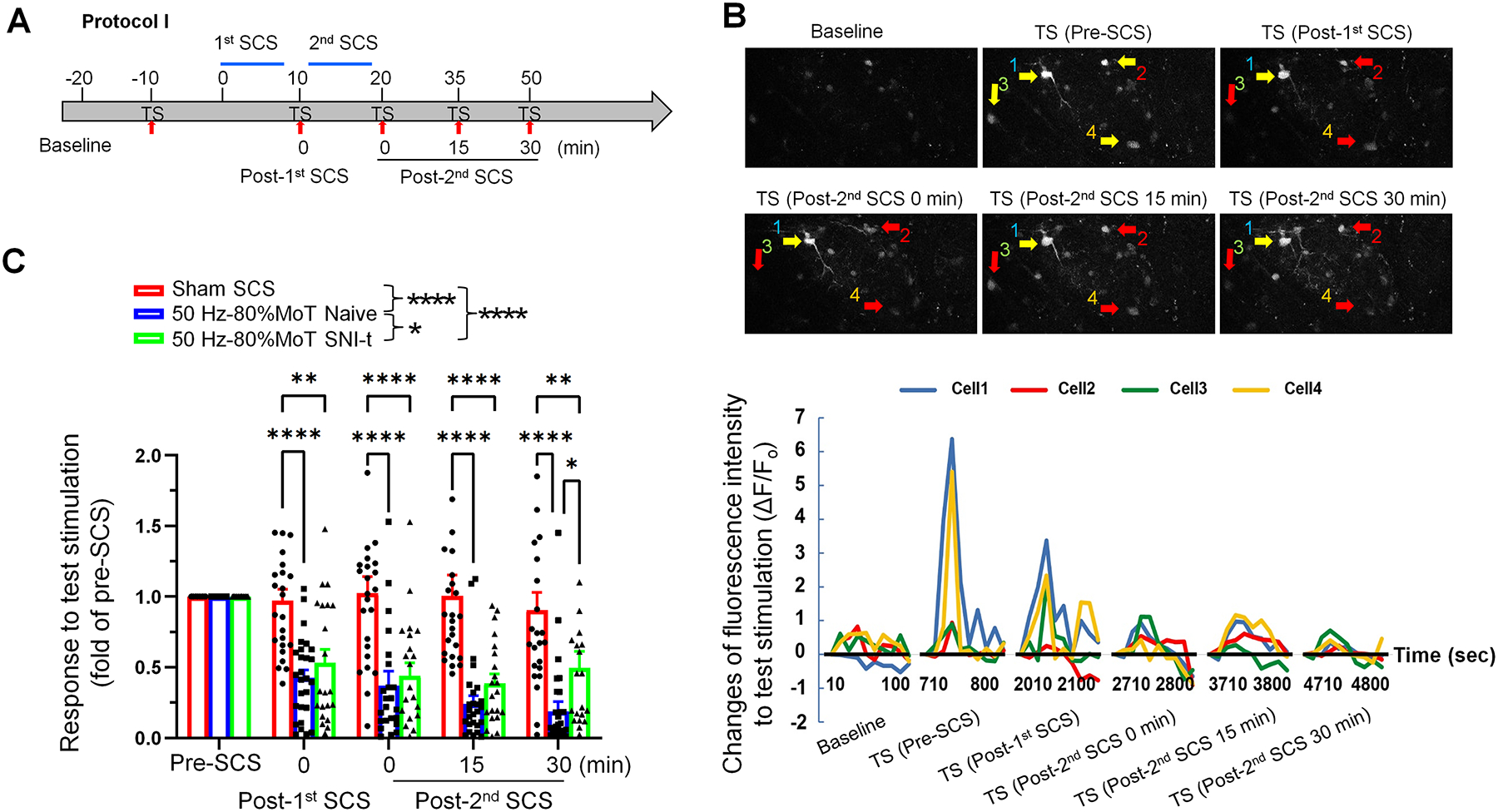
Short-term 50 Hz SCS at the high intensity inhibited calcium responses in superficial NK1R^+^ neurons to test stimulation in both naïve and SNI-t mice. **(A)** Illustration of the experimental procedure. After baseline imaging (background, 5 min), neuronal response to test stimulation (TS, 3.0 mA, 2.0 ms, 1 Hz, 10 sec, biphasic, constant current) was imaged at 10 min before SCS. Conventional SCS (50 Hz, 0.15 ms, 80% MoT) was then applied using Protocol I (two short sessions, 10 min/session) and neuronal responses to TS were then recorded at 0–5 min, 15–20 min, 30–35 min after completing SCS. **(B)** Upper: Examples of calcium responses in NK1R^+^ neurons to TS before and at different time points after SCS. Yellow arrows indicate neurons activated by TS. Red arrows indicated neurons activated by TS before SCS and also showed decreased responses to TS at post-SCS conditions. Lower: Fluorescence intensity traces (ΔF/F_o_) of four selected neurons to TS before and after SCS. **(C)** Conventional SCS significantly reduced response on superficial NK1R^+^ neurons after the 1^st^ session and from 0–30 min after the 2^nd^ session of SCS in both naïve (n=27 neurons from 4 mice) and SNI-t conditions (n=21 neurons from 6 mice), compared to that after sham SCS (n=24 neurons from 5 mice). The inhibition occurred rapidly after SCS, with a significant reduction observed at 0 min post-SCS, and lasted up to 30 min. The 2^nd^ SCS session induced a greater inhibition in naive mice than SNI-t mice at 30 min. Data are presented as mean ± SEM. *P<0.05, **P<0.01, ***P<0.001, ****P<0.0001, versus indicated group. Two-way mixed-model ANOVA with Bonferroni post hoc test.

**Figure 3.**
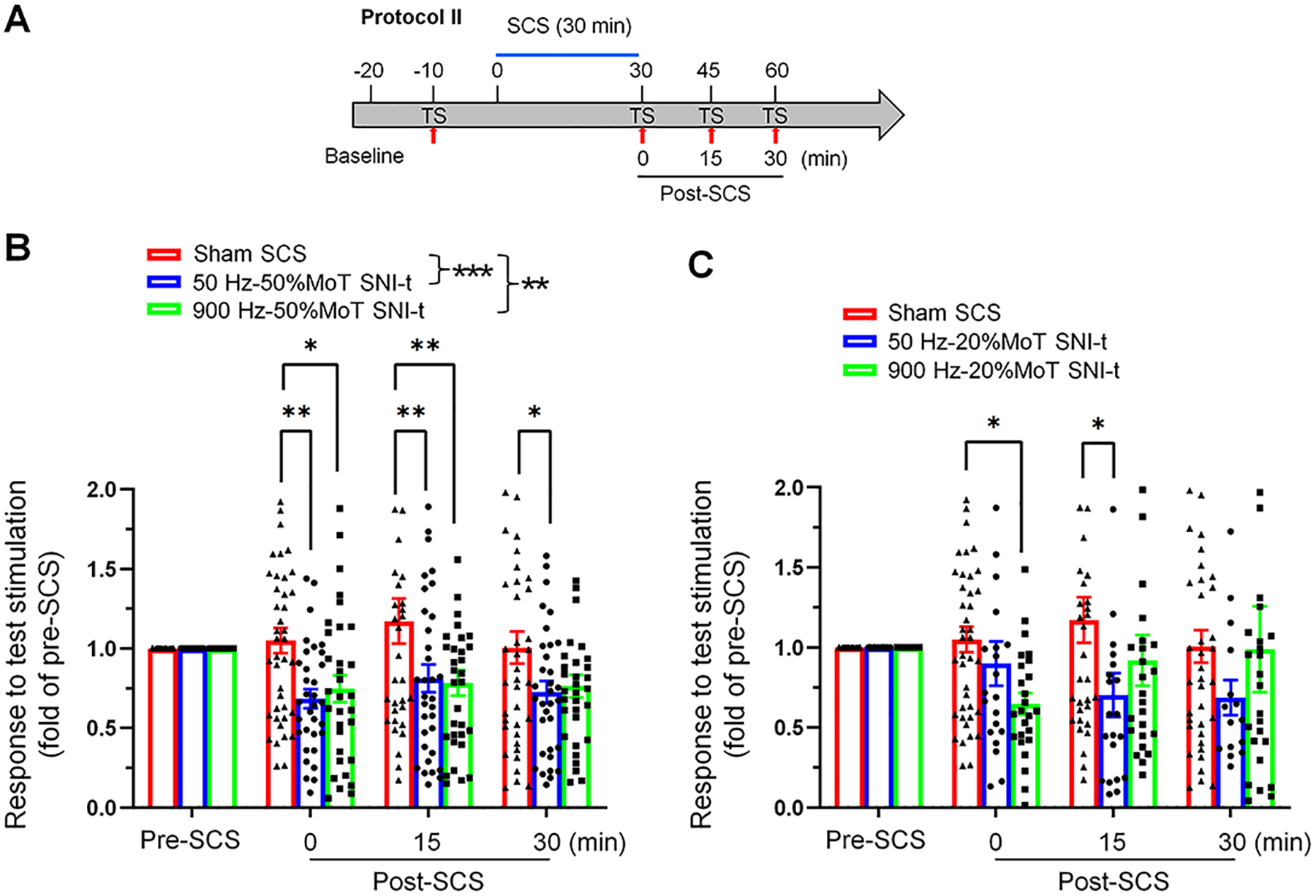
The inhibitory effects of 30-minute 50 Hz and 900 Hz SCS at the modest and low intensities on calcium responses of superficial NK1R^+^ neurons to test stimulation in SNI-t mice. **(A)** Illustration of the experimental procedure. After baseline imaging (background, 5 min), neuronal response to test stimulation (TS, 3.0 mA, 2.0 ms, 1 Hz, 10 sec, biphasic, constant current) was imaged at 10 min before SCS. SCS was then applied using Protocol II (one long session of 30 min), and neuronal responses to TS were then recorded at 0–5 min, 15–20 min, and 30–35 min after completing SCS. **(B)** Both 50 Hz (n=33 neurons from 5 mice) and 900 Hz (n=32 neurons from 5 mice) SCS applied for 30 min and the modest intensity (50% MoT) induced significant inhibitions on the response on superficial NK1R^+^ neurons to TS in SNI-t mice, compared to that after sham SCS (n=41 neurons from 8 mice). **(C)** 50 Hz (n=21 neurons from 5 mice) and 900 Hz (n=25 neurons from 5 mice) SCS applied for 30 min, but a low intensity (20% MoT) only mildly attenuated the response on superficial NK1R^+^ neurons in SNI-t mice, compared to that after sham SCS (n=41 neurons from 8 mice). Data are presented as mean ± SEM. *P<0.05, **P<0.01, ***P<0.001 versus indicated group. Two-way mixed-model ANOVA with Bonferroni post hoc test.

**Figure 4.**
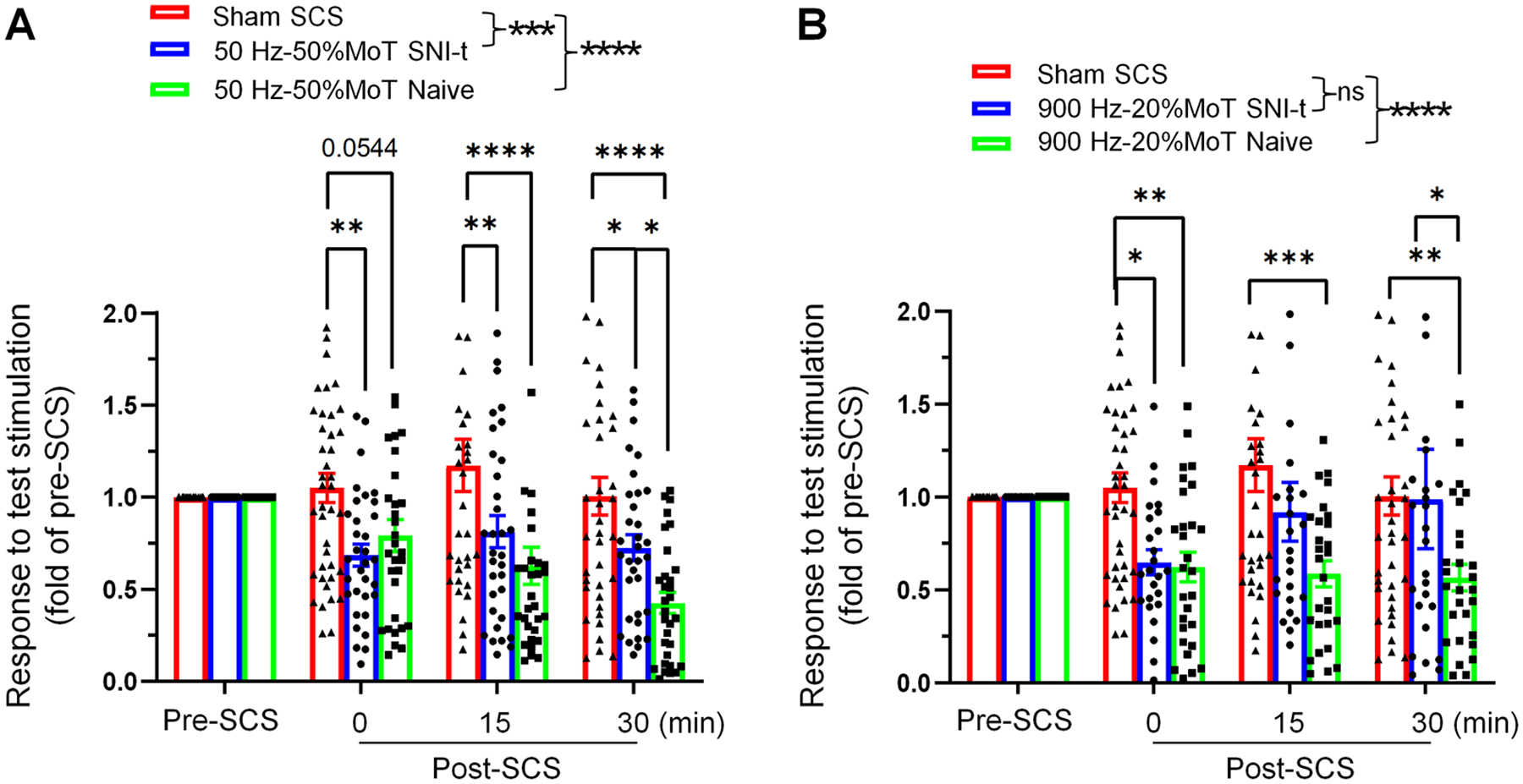
Comparing effects of 30-minute 50 Hz at the modest intensity and 900 Hz SCS at low intensity on the inhibition of superficial NK1R^+^ neurons to test stimulation between naïve and SNI-t mice. **(A)** Effects of 50 Hz SCS (50% MoT, Protocol II, 30 min) on the response on superficial NK1R^+^ neurons of in NK1R-Cre;GCaMP6s mice under naïve (n=31 neurons from 5 mice) and SNI-t (n=33 neurons from 5 mice) conditions, as compared to that after sham SCS (n=41 neurons from 8 mice). **(B)** Effects of 900 Hz SCS (20% MoT, Protocol II, 30 min) on the response on superficial NK1R^+^ neurons of in NK1R-Cre;GCaMP6s mice under naïve (n=28 neurons from 5 mice) and SNI-t (n=25 neurons from 5 mice) conditions, as compared to that after sham SCS (n=41 neurons from 8 mice). Data are presented as mean ± SEM. *P<0.05, **P<0.01, ***P<0.001, ****P<0.0001 versus indicated group. Two-way mixed-model ANOVA with Bonferroni post hoc test.

We randomized experiments to the different groups (e.g., naïve and SNI-t) and blinded the experimenter to the treatments (e.g., SCS paradigms) to reduce selection and observation bias. After the experiments were completed, no data point was excluded. Data were analyzed with the Prism 9.0 statistical program (GraphPad Software, Inc.). The statistical methods used for each study are described in the figure legends. For Figure 1, an unpaired t-test was done under a homoscedastic hypothesis, and for Figures 2, 3, and 4, a two-way mixed model analysis of variance (ANOVA) was performed, with one factor being the repeated measures (‘time’ factor: pre-SCS, 0 min post-SCS, 15 min post-SCS, 30 min post-SCS) and the other factor being the independent groups (e.g., different SCS paradigms or animal groups). The Bonferroni *post-hoc* test was used to compare the means of the groups at each time point. The ‘ns’ notation in the chart legends indicates a non-significant overall effect between the two groups based on the ANOVA analysis. The asterisks (*) above the bars indicate significant differences between the specific groups at that time point based on the *post-hoc* test results. Data are expressed as mean ± SEM. Two-tailed tests were performed, and *P* < 0.05 was considered statistically significant in all tests.

## 3 Results

### 3.1. 10-minute 50 Hz high-intensity SCS induced prolonged inhibition of superficial NK1R^+^ neurons in both naïve and SNI-t mice.

We first examined the effects of the low-rate (50 Hz) but high-intensity SCS (80% MoT), also known as conventional SCS[31; 41; 46; 58], on the inhibition of superficial NK1R^+^ neurons in naïve NK1R-Cre;GCaMP6s mice and in those approximately 2 weeks post-SNI-t. At this time point, neuropathic-pain like behavior is fully developed in this model[5; 13; 18; 27]. The baseline neuronal activity (F_o_) was imaged for a duration of 5 min, followed by imaging of evoked responses (F) to electrical TS at the ipsilateral hindpaw at 10 min pre-SCS (Fig. 1D). Evoked calcium signal amplitudes to TS, which were expressed as ΔF/F_o_, were greater in SNI-t mice than in naïve mice at pre-SCS conditions (Fig. 1E). Subsequently, two short 10-minute sessions of conventional SCS (50 Hz, 0.15 ms, 80% MoT) or sham SCS were applied, and a test stimulation was conducted between the sessions using Protocol I (Fig. 2A). The amplitudes of the evoked calcium responses to TS (ΔF/F_0_) were then measured at various time points after SCS and normalized to the value measured before the first session (pre-SCS, Fig. 2B,C). We observed a significant reduction in the calcium response to TS after SCS in both naïve and SNI-t mice, compared to sham SCS (Fig. 2C, Supplemental video). This inhibitory effect was observed quickly upon completion of the first SCS session. However, the second session, which was applied right upon completing the TS after the first session, did not result in an enhanced inhibitory effect than the initial session. The inhibitory effect following the second session persisted for 30 min in both naïve and SNI-t mice, suggesting a prolonged carry-over effect. Compared to the SNI-t mice, conventional SCS induced a greater reduction of the evoked calcium response to TS in naïve mice, indicating more pronounced inhibition (Fig. 2C).

### 3.2. 30-minute 900 Hz and 50 Hz SCS at moderate intensity also significantly inhibited NK1R^+^ neurons in SNI-t mice.

We further investigate how modification in the frequency, intensity, and treatment duration could alter the inhibition of superficial NK1R^+^ neurons by SCS. Considering that high-frequency SCS can induce pain inhibition at a lower intensity (e.g., < 50% MoT) than conventional SCS (80% MoT) in animal models[14; 41; 42], but may require a longer treatment duration[2; 21; 36], we applied 900 Hz SCS for 30 minutes (Protocol II, Fig. 3A) at either moderate (50% MoT) or low (20% MoT) intensities in SNI-t mice. For comparisons, 50 Hz and sham SCS were applied in the same manner, with each experiment testing only one SCS paradigm to avoid carry-over and time effects. At 50% MoT, both SCS paradigms significantly inhibited the responses in NK1R^+^ neurons to TS when compared to sham SCS (Fig. 3B). The inhibitory effects occurred quickly in both groups and persisted for 30 minutes. The overall level of inhibition and time courses were also comparable between the two groups.

### 3.3. 30-minute 900 Hz and 50 Hz SCS applied at low intensity only induced a transient and mild inhibition of superficial NK1R^+^ neurons in SNI-t mice.

In separate experiments, we further depicted the effects of 30-minute 900 Hz and 50 Hz SCS at a low intensity (20% MoT) in SNI-t mice. From group comparisons spanning 0–30 min, neither 900 Hz nor 50 Hz SCS resulted in a significant inhibition of superficial NK1R^+^ neurons when compared to sham SCS (Fig. 3C). Compared to sham SCS at the same time point, 900 Hz SCS induced a transient inhibition at 0 min post-SCS, while 50 Hz SCS led to a significant inhibition starting from 15 min post-SCS. Overall, the inhibitory effects were both mild and transient in these groups.

### 3.4. 30-minute 900 Hz SCS at low intensity induced less inhibition of superficial NK1R^+^ neurons in SNI-t mice than in naïve mice.

We observed a robust reduction of neuronal calcium response to TS following conventional SCS (10-minute, 50 Hz, 80% MoT), which effect was more pronounced in naive than in SNI-t mice (Fig. 2C). We further investigated whether this would also be observed with 30-minute SCS at moderate or low intensities. A 30-minute 50 Hz SCS at 50% MoT significantly inhibited the responses in superficial NK1R^+^ neurons to TS in both naïve and SNI-t groups, when compared to sham SCS (Fig. 4A). The inhibitory effects were quickly observed after SCS in both groups and persisted for 30 minutes. Except for 30 min-post SCS, there was no significant difference between naïve and SNI-t groups. At 20% MoT, 30-minute 900 Hz SCS did not induce significant neuron inhibition in SNI-t mice from group comparisons spanning 0–30 min (Fig. 4B), but induced a significant inhibition in naïve mice (Fig. 4B), revealing differential effects between naïve and neuropathic pain conditions.

## 4. Discussion

### 4.1. In vivo calcium imaging as an innovative approach for mechanistic study of SCS.

Based on the “gate control theory”[39; 50; 57], pain inhibition from conventional SCS was partly due to closing the “gate” by activating spinal inhibitory interneurons[19; 23; 50]. Approximately 80% of lamina I projection neurons are NK1R^+^[49; 54], which play a pivotal role in the spinal pain transmission[32; 45; 53]. Selective ablation of these neurons attenuated the development of inflammatory joint pain in rats[53], and reduced excitability of deeper dorsal horn neurons that are important for pain sensation and reflexes[44; 45]. However, the roles of NK1R^+^ neurons in the superficial dorsal horn in SCS-induced analgesia remain obscure. By using *in vivo* GCaMP imaging techniques, we could observe the diverse inhibitory actions that different SCS paradigms have on the responses of these neurons involved in pain transmission.

A previous histochemical study in NK1R-CreGFP mice showed that NK1R^+^ neurons were distributed in lamina I, III, and deeper laminae[57]. Notably, injecting a retrograde tracer at parabrachial (PB) nuclei revealed that ~58% of retrogradely labeled neurons in lamina I were NK1-CreGFP^+^, suggesting they are spinoparabrachial projection neurons[57]. With the development of novel NK1R-Cre;GCaMP6s mice, we can examine, for the first time *in vivo*, changes in NK1R^+^ neuronal response to SCS. This was achieved without the need for intra-spinal virus injection, which can lead to variable efficacy and distribution of GCaMP expression in different animals. This innovative approach may pave the way for new research opportunities into the role of these neurons in nociceptive processing and pain therapies.

### 4.2. The inhibition of superficial NK1R^+^ neurons presents an additional mechanism for pain inhibition by SCS.

Our results demonstrated that 50 Hz SCS at 80% MoT, which proven effective for pain inhibition in animal studies[31; 41; 46; 58], inhibited superficial NK1R^+^ neurons, some of which may be projection neurons of the spinal cord. Conventional SCS also inhibited wide-dynamic range (WDR) neurons and spinal local-field potentials (LFPs) evoked by C-fiber inputs in nerve-injured rats[26; 60; 62]. These findings collectively suggest that a broad inhibition of neurons involved in spinal nociceptive transmission may occur post-conventional SCS.

Consistent with the lasting neuron inhibitory effect post-SCS, previous studies showed that the suppression of neuropathic pain-like behavior by conventional SCS also exhibited a carry-over effect[14; 41; 61]. However, since we primarily used short-session SCS which may not accurately represent the extended SCS paradigms (e.g., spanning hours to days) employed in the clinic, it remains unclear whether longer SCS may have different effects on the reactivity of NK1R^+^ neurons, such as a greater carry-over effect. The detailed neurophysiological and neurochemical mechanisms of how SCS inhibits different types of dorsal horn neurons also warrant further investigation. The 900 Hz SCS at 80% MoT was not examined in the current study, as studies have shown that high-intensity SCS applied at frequencies approaching 800 Hz often induces unpleasant or painful sensations in patients[1; 50].

### 4.3. Different SCS paradigms result in variable degrees of inhibition in NK1R^+^ neurons.

Recent advancements have introduced new SCS paradigms (e.g., 10 kHz, DTM) to provide pain relief without causing paresthesia[2; 21; 36]. The amplitude of the epidurally recorded evoked compound action potential (ECAP) has been observed to increase with the SCS intensity[52]. This method has been used in clinical trials[33; 35; 51], and to examine dorsal column fiber activation by SCS in rats[10; 11; 17]. In rats, 50 Hz SCS at the effective ECAP intensity (~40% MoT) attenuated neuropathic pain-related behavior[17; 52]. In line with that finding, 30-minute 50 Hz and 900 Hz SCS at 50% MoT also significantly inhibited NK1R^+^ neurons in SNI-t mice. Accordingly, the inhibition of superficial NK1R^+^ neurons could partly contribute to pain reduction from SCS applied at the clinically relevant intensity.

The inhibitory effect from 900 Hz SCS at 50% MoT was comparable to that from 50 Hz, suggesting that a higher frequency may not be more effective in inhibiting NK1R^+^ neurons when applied at a moderate intensity. However, at 20% MoT which is below the sensory threshold[14; 43; 59; 60], 900 Hz SCS induced transient inhibition, whereas 50 Hz SCS only induced mild inhibition starting from 15 min post-SCS in SNI-t mice. Thus, different frequencies of SCS at a low intensity may exert different temporal effects on the inhibition of superficial NK1R^+^ neurons. Nevertheless, their overall inhibitory effects were much less than those applied at higher intensities, in line with previous observations that neuronal and pain inhibition by SCS were intensity-dependent[14; 41; 61].

Various substrates and mechanisms (e.g., superficial and deep dorsal horn neurons, supraspinal circuitry, glial cells) may be preferentially activated or modulated through different SCS paradigms to independently produce some degree of pain relief. The specifics of these mechanisms still require further investigation. Ultra-high frequency SCS (e.g., 10 kHz) produces analgesia without eliciting paresthesia. Intriguingly, increasing the frequency of SCS in patients eliminated both ECAP and paresthesia[37]. The hypothesis is that axons spike asynchronously when they are stimulated at a rate faster than their refractory period. These asynchronous spikes are unable to produce paresthesia because their transmission to the somatosensory cortex is blocked by feedforward inhibition[37]. The effects of new SCS paradigms on NK1R^+^ neurons, as well as their roles in pain inhibition, are areas that warrant future studies.

### 4.4. Nerve injury may compromise the neuronal inhibition induced by SCS.

SCS is effective for alleviating chronic pain of neuropathic origin, making animal models of neuropathic pain particularly suitable for exploring the underlying mechanisms[19; 23; 41]. Yet, the inhibitory effect of NK1R^+^ neurons from conventional SCS was more pronounced in naive mice than in SNI-t mice. Notably, the application of prolonged 900 Hz SCS at 20% MoT resulted in significant neuron inhibition in naïve but not in SNI-t mice. While we did not extend our comparison to the two groups subjected to 900 Hz SCS at 50% MoT, the insights gleaned from our current findings suggest differential impacts of 900 Hz stimulation under naive and neuropathic pain conditions. These findings also align with previous electrophysiology recording of eEPSCs in lamina II neurons, where there was a trend toward the inhibition of eEPSCs by 50 Hz electrical stimulation of peripheral Aβ-fibers being less pronounced in nerve-injured mice than in naïve mice[40].

Significant anatomical and neurochemical alterations occur in the spinal cord following injury, and dorsal horn neurons develop heightened responses to peripheral stimulation[8; 56]. Our *in vivo* GCaMP imaging also showed greater activation of NK1R^+^ neurons to TS in SNI-t mice than in naïve mice, which may partly contribute to the reduced inhibitory effect of SCS. Moreover, changes are observed in sensory mapping and excitatory synaptic transmission in lamina II. For example, the excitatory synaptic drive onto inhibitory neurons in the superficial dorsal horn may decrease after nerve injury, leading to reduced inhibition[30; 34]. The “gate control” theory postulates that SCS activates GABAergic inhibitory interneurons[23; 39; 57]. Yet, the endogenous GABAergic tone was found to decrease after nerve injury[30; 34]. Thus, pathological changes after nerve injury may compromise the mechanisms underlying the neuronal inhibition from SCS.

Our findings of attenuated NK1R^+^ neuron responsiveness following SCS in naïve mice raise intriguing questions about its potential functional relevance. There is scant evidence from animal or human studies concerning the modulation of nociceptive pain in physiological conditions by SCS. Nonetheless, SCS was shown to inhibit WDR neuron responses in naïve rats[22; 35], and the activation of low-threshold afferent fibers also attenuated high-threshold C-fiber-evoked ESPCs in naïve mice[40]. Future studies incorporating behavioral assessments of pain sensitivity in naïve animals, along with clinical studies in healthy human subjects, could provide valuable insights into the potential broader effects of SCS on pain processing.

There are some technical limitations to consider. Firstly, in an effort to better standardize repeated stimulation (e.g., consistent intensity and pattern), we utilized electrical test stimulation. However, this may not fully emulate the physiological mechanisms involved in the transduction of natural mechanical or thermal stimuli. Future studies that incorporate biologically relevant stimuli could yield further insights into the modulation of superficial NK1R^+^ neurons by SCS under physiologically relevant conditions. Secondly, due to the slow dynamic of calcium signals, repeated stimulation is often employed to evoke neuronal responses *in vivo*[3; 20; 47; 57]. However, as repeated stimulation can induce activity-dependent neuronal sensitization, such as windup, in dorsal horn neurons[4; 24; 25; 55], the calcium responses in NK1R^+^ neurons to 1 Hz test stimulation may reflect both the activation and sensitization of their activity, effects that can be mitigated by SCS. Thirdly, the incorporation of new techniques such as 10 kHz and DTM SCS could offer insights into the mechanisms of action of these clinical paradigms[21; 29; 46; 58]. Lastly, additional research is needed to examine potential sex differences and fully comprehend why the efficacy of pain control with SCS decreases over time[12; 39].

## Conclusions

Our findings suggest a new neuronal mechanism that contributes to the pain-inhibitory effects of SCS. The use of *in vivo* GCaMP imaging offers a promising avenue for advancing mechanistic study of different SCS paradigms and optimization SCS-induced analgesia.

## Supplementary Material

Supplementary Materials: movies, audio **Supplemental Video.** An example of SCS (50 Hz, 80% MoT, Protocol I) inhibited the calcium responses in superficial NK1R^+^ neurons to the electrical test stimulation (TS, 3.0 mA, 2.0 ms, 1 Hz, 10 seconds, biphasic, constant current) in a SNI-t mouse.

Supplementary Materials: figures

## Figures and Tables

**Table 1.** Parameters of different SCS paradigms include stimulation frequency, pulse width (PW), amplitude, and duration of stimulation. Sham, sham SCS.

Frequency (Hz)	Pulse width (ms)	Amplitude (MoT)	Duration (min)	Animal condition and group size
50	0.15	80%	10	SNI-t (n=6 mice, 21 neurons)
50	0.15	50%	30	SNI-t (n=5 mice, 33 neurons)
50	0.15	20%	30	SNI-t (n=5 mice, 21 neurons)
900	0.15	50%	30	SNI-t (n=5 mice, 32 neurons)
900	0.15	20%	30	SNI-t (n=5 mice, 25 neurons)
50	0.15	80%	10	Naive (n=4 mice, 27 neurons)
50	0.15	50%	30	Naive (n=5 mice, 31 neurons)
900	0.15	20%	30	Naive (n=5 mice, 28 neurons)
Sham	0	0	10	Naive/SNI-t (n=5 mice, 24 neurons)
Sham	0	0	30	Naive/SNI-t (n=8 mice, 41 neurons)

## Data Availability

Data sets that are generated from the current study will be available and shared with all communities of scientists upon request.
