# Supplementary material for: Visualizing the modulation of neurokinin 1 receptor-positive neurons in the superficial dorsal horn by spinal cord stimulation in vivo
Source: Pain. Author manuscript; Available in PMC 2025 Apr 24. (PMC11723817; doi:10.1097/j.pain.0000000000003361)
Supplement: Supplementary Materials: figures [file NIHMS2006493-supplement-Supplementary_Materials__figures.pdf]

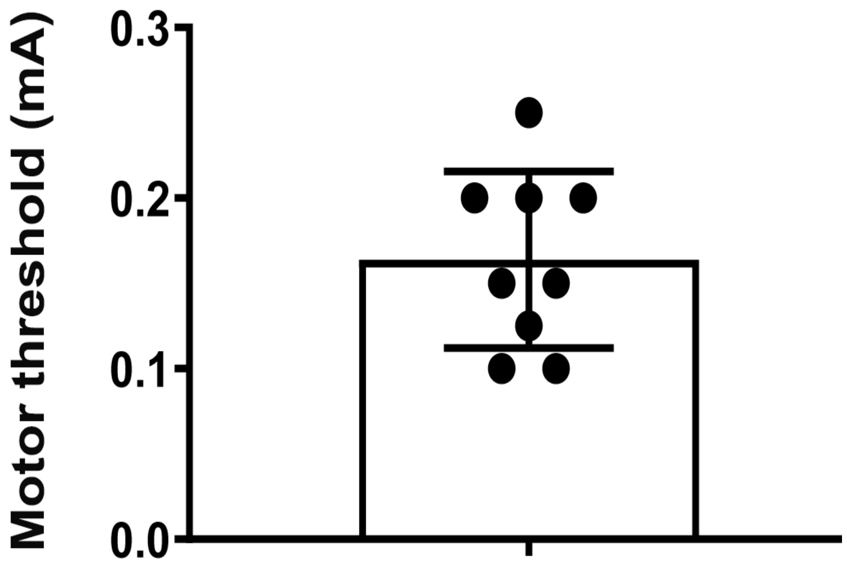

Supplemental Figure 1. The example of motor thresholds measured in a subgroup of anesthetized mice (N=9) to 4 Hz spinal cord stimulation (0.15 ms pulse width, biphasic, constant current). Data are presented as mean ± SEM.
